# Development and external validation of a three-stage model to predict live birth after fresh IVF/ICSI embryo transfer

**DOI:** 10.3389/fendo.2026.1916182

**Published:** 2026-09-21

**Authors:** Haoxuan Guo, Yanhui Liu, Juncheng Tong, Xiaoke Zhang, Fanggui Wu, Sheng Yang, Sibo Zhang, Nanxiong Wang, Xibu Wang, Jianting Luo, Shangjie Liu, Kai Zhao, Mengyuan Qu

**Affiliations:** 1 The Reproductive Medical Center, The Third Affiliated Hospital of Shenzhen University, Shenzhen Luohu People’s Hospital, Shantou University Medical College, Shenzhen, China; 2 School of Life Science and Technology, Wuhan Polytechnic University, Wuhan, China; 3 Department of Reproductive Medicine, The Third Affiliated Hospital of Zhengzhou University, Zhengzhou, China; 4 Institute of Reproductive Health, Tongji Medical College, Huazhong University of Science and Technology, Wuhan, China

**Keywords:** external validation, IVF (ICSI), live birth, machine learning, predictive model

## Abstract

**Background:**

Predicting live birth outcomes following *in vitro* fertilization/intracytoplasmic sperm injection (IVF/ICSI) remains challenging. Most existing models are typically limited to single time-point data and lack integration of sequential clinical information, constraining their utility for personalized decision-making.

**Methods:**

This retrospective multicenter study included 8,389 fresh IVF/ICSI cycles for model development and internal validation, with 2,058 cycles for independent external validation. We constructed prediction models at three sequential clinical stages: Stage 1 (baseline), Stage 2 (ovarian stimulation), and Stage 3 (embryo transfer). We compared 10 base machine learning models and 3 ensemble methods (SES, Stacking, DEP). Performance was evaluated using area under the receiver operating characteristic curve (AUC-ROC), net reclassification improvement (NRI), integrated discrimination improvement (IDI), calibration, and decision curve analysis (DCA). Model interpretability was assessed via SHapley Additive exPlanations (SHAP) analysis and restricted cubic splines (RCS).

**Results:**

The three-stage sequential model showed stepwise improved predictive performance. In internal validation, the SES ensemble achieved AUC increases from 0.699 (Stage 1) to 0.754 (Stage 3). In the test cohort, AUC improved significantly from 0.678 at baseline to 0.725 after ovarian stimulation (NRI = 0.630, IDI = 0.072, P<0.001), with a modest further increase to 0.731 at the embryo development stage. External validation yielded moderate discriminative performance (Stage 3 AUC = 0.719). DCA showed favorable clinical net benefit. SHAP analysis identified female age and high-quality embryos transferred as the dominant predictors. RCS confirmed nonlinear associations of female age, ovarian reserve, endometrial thickness, oocyte yield and embryo quantity metrics with live birth.

**Conclusions:**

This three-stage sequential, externally validated model provides reliable and interpretable live birth prediction at key decision points during fresh IVF/ICSI treatment. It may support personalized pretreatment counseling. However, prospective validation and predefined clinical thresholds are required prior to routine clinical decision-making.

## Introduction

Globally, infertility affects approximately 17.5% of individuals during their lifetime, representing a serious medical and psychosocial burden (1). Assisted reproductive technologies, particularly *in vitro* fertilization (IVF) and intracytoplasmic sperm injection (ICSI), have revolutionized the treatment of infertility, offering hope to millions of couples worldwide. Despite continuous advances in laboratory techniques and clinical protocols, the live birth rates per cycle remain relatively modest at 30–40% and decline significantly with age. The complexity of IVF/ICSI outcomes arises from multiple interacting factors across the treatment continuum, including patient demographics, ovarian reserve, hormonal dynamics, stimulation protocols, embryological characteristics, and endometrial receptivity (2, 3). Predicting which patients will achieve a live birth remains a clinical challenge.

Traditional prediction tools, such as morphological embryo grading and logistic regression models, are limited by subjectivity, linear assumptions, and an inability to capture complex interactions (4). Most existing models operate at a single time point—either before treatment or after embryo transfer, and fail to integrate information as it accumulates along the clinical pathway (5). While some studies have developed two-stage or multi-phase models, their performance has been modest, and few focus specifically on live birth (6, 7). There is a clear need for a dynamic, multi-stage framework that reflects how clinical decisions are actually made: initial assessment, stimulation monitoring, and embryo transfer decision-making.

Machine learning offers new tools for integrating high-dimensional clinical data. Recent studies have shown promise: Borji et al. developed a TabTransformer-based deep learning pipeline on large-scale Human Fertilization and Embryology Authority dataset (8); Zhang et al. applied artificial neural networks to predict live birth in natural-cycle IVF (9); and Sadegh-Zadeh et al. achieved 96.35% accuracy using ensemble methods (10). However, most existing models are single-staged, lack rigorous external validation, or treat machine learning as a black box. Few studies have systematically compared basic learners with advanced ensemble strategies or validated models across independent cohorts. Equally important, model interpretability and clinically actionable threshold identification have rarely been explored, which substantially limits clinical translation and real-world application.

In this study, we developed a three-stage machine learning pipeline for live birth prediction at key clinical decision points: baseline, ovarian stimulation, and embryo transfer. Ten base models and three ensemble strategies were evaluated using internal and external validation. We used SHapley Additive exPlanations (SHAP), restricted cubic splines (RCS), and decision curve analysis (DCA) to build a transparent, dynamic, and clinically applicable tool for personalized IVF/ICSI assessment and patient counseling.

## Methods

### Study design and population

This retrospective study included 8,389 women undergoing their first fresh IVF/ICSI cycles at a university-affiliated reproductive center in Shenzhen, China from January 2019 to December 2024. A temporal external validation cohort comprising 2,058 patients undergoing their first fresh cycles was enrolled from another independent tertiary hospital in Wuhan, China between June 2024 and June 2025. Data were retrieved from standardized electronic medical records at both centers. Inclusion criteria were: female age 20–45 years; fresh embryo transfer; available follow-up data for the primary outcome. Exclusion criteria were: cycles cancellation before oocyte retrieval or embryo transfer; use of donor gametes; missing live birth outcome; preimplantation genetic testing (PGT) cycles. The primary outcome was live birth, defined as delivery of at least one live infant after 24 weeks of gestation. Multiple pregnancies resulting in at least one live birth were counted as a single live-birth event. The study was approved by the Research Ethics Committee of each hospital. Due to the retrospective, anonymized nature of the study, informed consent was waived.

The dataset was split into three mutually exclusive subsets by stratified random sampling to preserve the live birth distribution. The training set (70%, n=5871) was used for model training, feature engineering, and internal cross-validation. The internal validation set (15%, n=1259) was used for monitoring training, early stopping, hyperparameter optimization, Platt scaling, and dynamic pruning. The test set (15%, n=1259) was a fully locked unseen dataset for final model evaluation. Flowchart of this study is shown in **Figure 1**.

**Figure 1:**
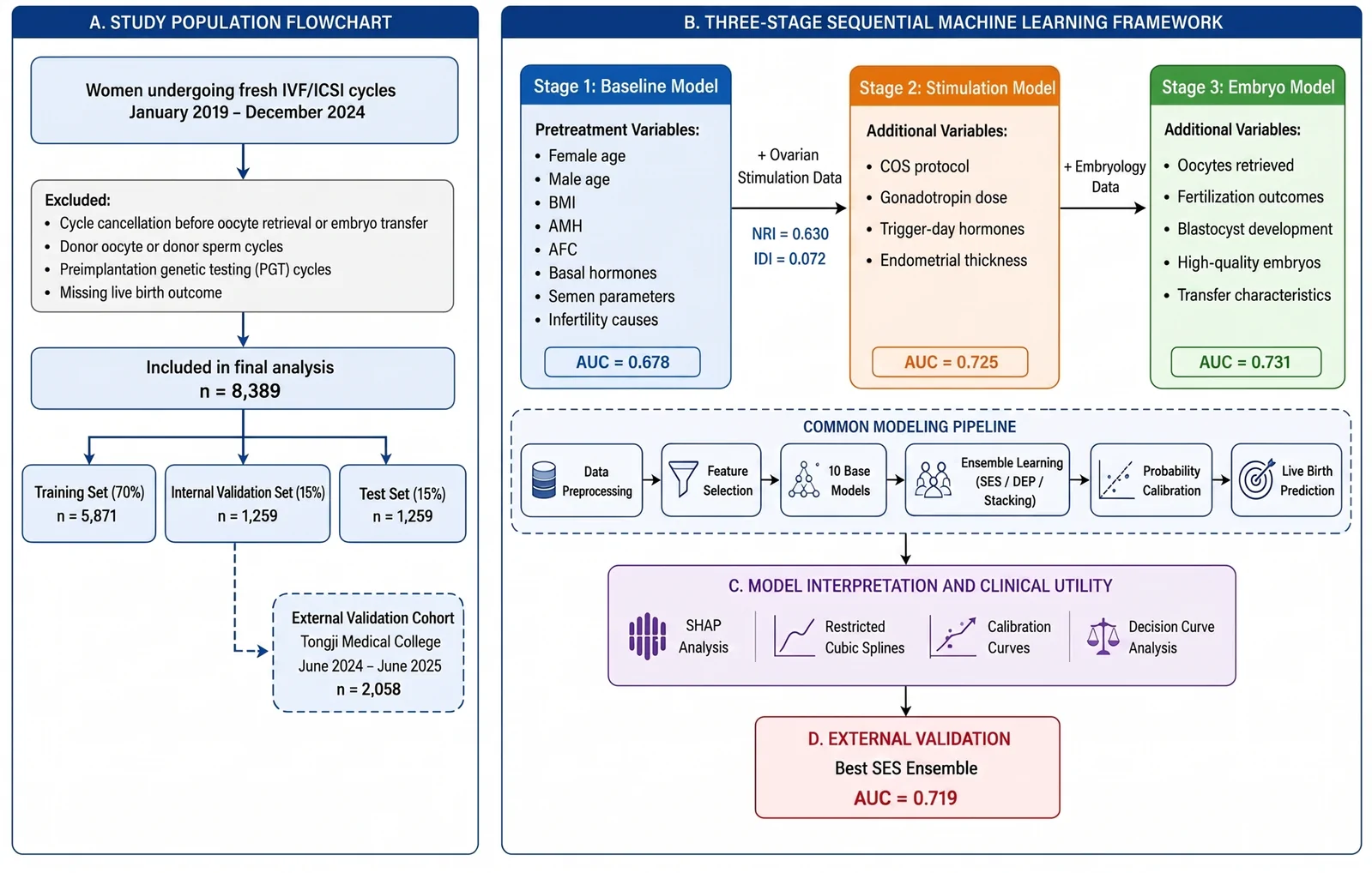
Study flowchart and three-stage sequential machine learning framework.

To mimic real-world clinical workflow and prevent look-ahead information leakage, a cumulative staging strategy was applied. Three sequential clinical stages were defined: Stage 1 (baseline stage): Variables available at initial consultation, including demographics, infertility causes and type, baseline hormones on day 2–3 of the menstrual cycle, and semen parameters. Ovulatory disorder (female factor 3) was defined according to the Rotterdam criteria, including polycystic ovary syndrome (PCOS), hypothalamic hypogonadism, hyperprolactinemia, and other causes of oligo-ovulation or anovulation. Diminished ovarian reserve (DOR, female factor 4) was defined per the Bologna criteria; advanced maternal age in this study referred to female age ≥38 years at oocyte retrieval. Stage 2 (stimulation stage): All Stage 1 variables plus ovarian stimulation protocol, gonadotropin dosage, trigger day hormone levels, and endometrial thickness (EMT). Stage 3 (embryo development stage): All Stage 2 variables plus oocyte yield, fertilization, embryo development metrics, and embryo characteristics. Variables acquired in later stages were not introduced into earlier-stage models. Fresh transfer included Day-3 cleavage embryos or Day-5 blastocysts. High-quality Day 3 embryos were defined as 7–9 blastomeres, ≤10% fragmentation, and no multinucleation. High-quality blastocysts were defined as ≥3BB by Gardner grading.

### Data preprocessing

A rigorous leakage-proof preprocessing framework was established. All continuous variables were winsorized at the 1st and 99th percentiles to reduce outlier effects. Overall missingness was low across all predictors, and all missing values were imputed using MissForest trained exclusively on the training set. Missing data were imputed after dataset splitting under a strict stage-isolation strategy. Continuous missing values were imputed by the MissForest algorithm (n_estimators=30, max_iter=5); categorical variables were imputed by mode. The imputation model was trained only on the training set and applied to validation and test sets with a fixed random seed (42). All preprocessing steps were fitted exclusively on the training set to prevent data leakage. Variables with variance inflation factor (VIF) > 10 were removed to address multicollinearity. A dual preprocessing pipeline was used: For tree-based models (RF, XGBoost, LightGBM, CatBoost), variables were retained in their original scales, and class imbalance was handled via class weight balancing. For linear models and neural networks, continuous variables were normalized using the RobustScaler and transformed with the Yeo-Johnson transformation to reduce skewness, and categorical variables were one-hot encoded. Given the near-balanced outcome distribution, no oversampling was applied.

### Feature selection

Feature selection was performed exclusively on the training set using a multi-algorithm voting framework: 1. LASSO regression with 5-fold cross-validation, selecting variables with non-zero coefficients using the minimum λ criterion. 2. Random forest feature importance (n_estimators = 100) to rank variables by mean decrease in impurity. 3. Recursive feature elimination (RFE) with a logistic regression estimator to iteratively remove low-impact features. 4. Boruta algorithm (max_depth = 7) to identify features significantly more important than random shadow variables. Features supported by at least two algorithms, or included in the predefined clinical whitelist (clinical consensus), were retained for subsequent modeling. Selection results of key variables were shown in **Supplementary Table 2**.

### Model construction and ensemble framework

Ten machine learning models were trained: Logistic Regression (LR), Random Forest (RF), XGBoost, LightGBM, CatBoost, Support Vector Machine (SVM), Multilayer Perceptron (MLP), Naive Bayes (NB), K-Nearest Neighbors (KNN), and Decision Tree (DT). Hyperparameter optimization was performed in Optuna with the Tree-structured Parzen Estimator (TPE) and 30 trials, using 5-fold stratified cross validation with area under the receiver operating characteristic curve (AUC-ROC) as the objective. Early stopping was applied with a patience of 100 rounds. Platt scaling was conducted on the internal validation set to calibrate predicted probabilities. A three-dimensional ensemble strategy was implemented: 1. Static Ensemble Selection (SES): NSGAII algorithm (population=30, generations=20) selected a Pareto-optimal subset of base learners by minimizing prediction error and inter-model correlation. 2. Dynamic Ensemble Pruning (DEP): For each test sample, 15 nearest neighbors were identified in the internal validation set using Euclidean distance. The worst 50% of models were dynamically pruned, and average predictions from the top-performing subset were used. 3. Stacking: A cross-validated logistic regression served as the meta-learner to combine pruned base model predictions. All models were compared in the internal validation set. The best-performing base model and three ensemble strategies were evaluated in the test and external validation cohorts.

### Model evaluation

Model performance was assessed using AUC-ROC, precision-recall curve (PR-AUC), accuracy, sensitivity, specificity, Brier score, positive predictive value (PPV), negative predictive value (NPV) and F1 score. Calibration was evaluated by calibration curves. Clinical utility was assessed by DCA across threshold probabilities of 1%–99% against treat-all and treat-none strategies. Bootstrapping with 1000 replicates was used to estimate 95% confidence intervals. The DeLong test was used for paired AUC comparisons. Net reclassification improvement (NRI) and integrated discrimination improvement (IDI) were used to quantify incremental gains across stages. Subgroup analyses were stratified by infertility cause, type, female age, Body Mass Index (BMI), and polycystic ovary syndrome (PCOS) status, with interaction *P*-values to test heterogeneity.

### Interpretability analysis

To interpret the complex SES-DEP ensemble model, surrogate model distillation was first performed: a shallow XGBoost regressor (max_depth = 4, learning_rate = 0.05) was trained to approximate the ensemble’s predictions. A pre-specified threshold of surrogate-model R² > 0.8 was planned for proceeding to SHAP analysis and to quantify the contribution of each feature to individual predictions (Tree-SHAP). Mean absolute SHAP values were used to rank global feature importance. SHAP dependence plots were used to illustrate directional effects. RCS regression was used to evaluate the nonlinear association between continuous variables and live birth, with knots at the 5th, 35th, 65th, and 95th percentiles. All RCS models were strictly adjusted for major clinical confounders. Subgroup RCS curves were conducted stratified by female age at 35 years. P for overall association and P for non-linearity were calculated to evaluate linear and non-linear trends.

### Statistical analysis and software

Continuous variables are presented as median with interquartile range (IQR) and compared using the Wilcoxon rank-sum test. Categorical variables are summarized as counts and percentages and compared using the chi-squared test or Fisher’s exact test. All machine learning analyses were performed using Python version 3.9. Data preprocessing was implemented using scikit-learn and imbalanced-learn. Hyperparameter optimization was performed using Optuna with TPE, and SES was implemented using DEAP. Statistical analyses and visualizations were performed using SciPy, Statsmodels, SHAP, Matplotlib, and Seaborn. All statistical tests were two-sided, and a P value < 0.05 was considered statistically significant.

## Results

### Study population characteristics

**Figure 1** showed the flowchart of this study. A total of 10,447 fresh IVF/ICSI cycles were included: 5,871 in training, 1,259 in internal validation, 1,259 in testing, and 2,058 in external validation. Live birth rates were 42.3%, 44.4%, 43.4%, and 44.7%, respectively. Baseline characteristics were shown in **Table 1**. Most baseline characteristics were well balanced among the internal cohorts (all P > 0.05), confirming effective stratified randomization. Within the training cohort, women achieving live birth were significantly younger (median age 32 vs. 35 years, P < 0.001), with higher ovarian reserve indicated by higher anti-Müllerian hormone (AMH) and antral follicle count (AFC). Male partners in the live birth group were also younger with lower sperm DNA fragmentation index (DFI). The live birth group had a higher proportion of ovulatory disorders and a lower rate of DOR or advanced maternal age (both P < 0.001).

**Table 1:** Baseline demographic and clinical characteristics of the study population across four cohorts.

Characteristic	Overall (n=10447)	Training cohort			Internal validation (n=1259)	P	Testing cohort (n=1259)	P	External validation (n=2058)	P
		Non-live birth (n=3386)	Live birth (n=2485)	P						
Demographics										
Female Age, years	33 [30, 37]	35 [31, 39]	32 [30, 35]	**<0.001**	33 [30, 37]	0.133	33 [30, 37]	0.449	32 [29, 36]	**<0.001**
Male Age, years	35 [32, 39]	36 [32, 40]	34 [31, 37]	**<0.001**	35 [32, 39]	0.824	35 [32, 39]	0.851	35 [32, 38]	0.040
Female BMI	21.63 [19.95, 23.56]	21.77 [20.00, 23.88]	21.63 [19.92, 23.63]	0.066	21.58 [19.91, 23.62]	0.600	21.63 [19.89, 23.50]	0.182	21.44 [19.98, 23.05]	**<0.001**
Female Weight	54.36 [51.62, 57.48]	54.64 [51.96, 57.62]	54.50 [51.76, 57.60]	0.242	54.54 [51.96, 57.48]	0.980	54.48 [51.84, 57.42]	0.365	53.45 [50.39, 57.00]	**<0.001**
Infertility duration, years	3 [2, 6]	3 [2, 6]	3 [2, 5]	**0.005**	3 [2, 5]	0.091	3 [2, 6]	0.877	3 [2, 6]	0.213
Infertility type, n (%)			**<0.001**		0.588		0.393		0.045	
Primary infertility	4340 (41.5)	1297 (38.3)	1128 (45.4)		509 (40.4)		503 (40.0)		903 (43.9)	
Secondary infertility	6107 (58.5)	2089 (61.7)	1357 (54.6)		750 (59.6)		756 (60.0)		1155 (56.1)	
Infertility causes, n (%)										
Tubal/peritoneal factor	6349 (60.8)	1977 (58.4)	1518 (61.1)	0.040	751 (59.7)	0.962	766 (60.8)	0.407	1337 (65.0)	**<0.001**
Endometriosis	1249 (12.0)	411 (12.1)	338 (13.6)	0.105	150 (11.9)	0.441	162 (12.9)	0.953	188 (9.1)	**<0.001**
Ovulatory disorder	1386 (13.3)	345 (10.2)	373 (15.0)	**<0.001**	159 (12.6)	0.731	145 (11.5)	0.512	364 (17.7)	**<0.001**
Advanced age/DOR	2704 (25.9)	1184 (35.0)	432 (17.4)	**<0.001**	333 (26.4)	0.458	313 (24.9)	0.058	442 (21.5)	**<0.001**
Unexplained	1495 (14.3)	487 (14.4)	396 (15.9)	0.108	181 (14.4)	0.578	195 (15.5)	0.719	236 (11.5)	**<0.001**
Male Factor	5068 (48.5)	1701 (50.2)	1241 (49.9)	0.368	632 (50.2)	0.890	620 (49.2)	0.740	874 (42.5)	**<0.001**
Baseline hormones										
FSH, IU/L	6.83 [5.70, 8.30]	7.04 [5.89, 8.63]	6.83 [5.76, 8.13]	**<0.001**	6.92 [5.78, 8.48]	0.935	6.99 [5.92, 8.42]	0.486	6.30 [5.06, 7.70]	**<0.001**
LH, IU/L	4.49 [3.21, 6.14]	4.61 [3.31, 6.29]	4.70 [3.36, 6.39]	0.048	4.70 [3.39, 6.34]	0.872	4.70 [3.45, 6.43]	0.227	3.77 [2.68, 5.25]	**<0.001**
FSH/LH	1.56 [1.14, 2.15]	1.59 [1.15, 2.21]	1.48 [1.07, 2.02]	**<0.001**	1.53 [1.13, 2.12]	0.933	1.53 [1.13, 2.12]	0.698	1.66 [1.23, 2.28]	**<0.001**
E2, pg/mL	36.00 [27.00, 50.00]	37.00 [27.00, 53.00]	36.00 [27.00, 48.00]	**0.007**	36.00 [25.81, 53.06]	0.519	35.29 [26.00, 50.00]	0.101	36.46 [30.00, 46.96]	**0.012**
PRL, ng/ml	15.75 [11.23, 22.31]	15.68 [11.04, 21.94]	15.92 [11.42, 22.59]	0.035	16.08 [11.54, 22.61]	0.208	16.25 [11.32, 23.43]	0.115	15.26 [11.04, 21.56]	0.060
T, ng/ml	0.25 [0.16, 0.38]	0.24 [0.14, 0.36]	0.25 [0.16, 0.38]	**0.010**	0.24 [0.15, 0.39]	0.424	0.23 [0.15, 0.37]	0.471	0.29 [0.19, 0.39]	**<0.001**
AMH, ng/mL	2.46 [1.39, 3.99]	2.19 [1.18, 3.63]	2.67 [1.58, 4.22]	**<0.001**	2.46 [1.35, 4.03]	0.332	2.49 [1.49, 3.86]	0.146	2.70 [1.57, 4.28]	**<0.001**
AFC, n	11 [7, 16]	10 [6, 15]	12 [8, 18]	**<0.001**	11 [7, 16]	0.356	11 [7, 16]	0.410	13 [8, 19]	**<0.001**
Sperm parameters										
Progressive motility, %	46.00 [39.00, 52.96]	45.80 [39.40, 52.20]	46.00 [39.20, 52.40]	0.817	46.00 [39.40, 52.50]	0.650	45.80 [38.80, 52.38]	0.449	47.00 [37.00, 56.00]	**0.007**
Concentration, 10^6^/mL	56.52 [42.84, 73.00]	56.18 [43.40, 71.32]	58.03 [44.78, 74.09]	**<0.001**	56.62 [43.82, 72.95]	0.684	56.96 [44.19, 72.13]	0.718	53.29 [35.92, 75.65]	**<0.001**
Abnormal Sperm, %	96.60 [95.40, 97.60]	96.60 [95.60, 97.60]	96.60 [95.40, 97.60]	0.364	96.60 [95.40, 97.60]	0.967	96.60 [95.59, 97.60]	0.979	97.00 [95.20, 98.20]	**<0.001**
DFI, %	19.69 [16.34, 24.00]	20.09 [16.61, 24.21]	19.44 [16.07, 23.47]	**<0.001**	19.76 [16.53, 24.04]	0.775	19.90 [16.62, 24.12]	0.310	19.09 [15.18, 24.29]	**<0.001**
HDS, %	8.69 [7.21, 10.52]	8.77 [7.29, 10.57]	8.65 [7.19, 10.42]	0.132	8.75 [7.36, 10.34]	0.685	8.64 [7.28, 10.50]	0.867	8.55 [6.83, 10.76]	**0.020**

Data are presented as median [interquartile range] for continuous variables and n (%) for categorical variables. *P-value comparing training, internal, external validation, and testing cohorts; †P-value comparing live birth and non-live birth groups in the training cohort. *DOR, diminished ovarian reserve; BMI, body mass index; FSH, follicle-stimulating hormone; LH, luteinizing hormone; E2, estradiol; PRL, prolactin; AMH, anti-Müllerian hormone; AFC, antral follicle count; DFI, DNA fragmentation index. HDS, high DNA stainability.

The bold values in **Table 1** indicate statistically significant differences (P < 0.05).

Ovarian stimulation, embryology, and transfer parameters are presented in **Supplementary Table 1**. The live birth group more commonly underwent the long GnRH agonist protocol, had thicker trigger-day endometrium, more oocytes retrieved, more MII oocytes, and more 2PN zygotes (all P < 0.001). Blastocyst formation rate, blastocyst count, cleavage embryos, and frozen embryos were all significantly higher (all P < 0.001). Patients with live birth also had higher rates of day 5 blastocyst transfer and single high-quality embryo transfer (both P < 0.001). The external validation cohort showed expected differences in several baseline and treatment variables, providing a geographically and temporally independent dataset for robust external validation.

### Feature selection results

We used a multi-algorithm voting framework including LASSO, RF, RFE, Boruta, and clinical priority evaluation to identify key predictors across the three sequential stages. For each stage, LASSO regression was used for dimensionality reduction, with optimal penalty parameters (α) validated via MSE paths, and coefficient shrinkage at the best α confirmed stable feature elimination. RF Gini importance plots verified feature relevance across stages (**Supplementary Figure 1**). The algorithm intersection network (**Supplementary Figure 2**) visualized the consensus features for each stage, including clinically meaningful predictors such as female age, trigger-day progesterone, high-quality embryos transferred, and EMT, confirming clinical plausibility. Detailed voting scores are shown in **Supplementary Table 2**. Variables were retained if selected by at least two algorithms or judged to be clinically indispensable (**Supplementary Table 3**).

### Model performance across three stages

Stage-specific prediction models were developed and evaluated in internal validation and test cohort. In internal validation cohort, 10 base models (KNN, RF, CatBoost, LightGBM, XGBoost, NB, SVM, LR, MLP, DT) were compared (**Supplementary Figures 3A–D**). The best-performing model was then compared with three ensemble strategies (SES, Stacking, and DEP ensemble). The SES ensemble displayed progressive AUC improvement from Stage 1 (0.699) to Stage 2 (0.723) and Stage 3 (0.754). The ensemble methods outperformed the best base model, with detailed performance shown in **Supplementary Figures 4A–D**.

In the test cohort (**Figure 2A**, **Table 2**), stacking ensemble model yielded highest AUC of 0.678 (95% CI: 0.649-0.708) at the baseline stage. The stimulation stage model achieved an AUC of 0.725 (95% CI: 0.698–0.751), representing a significant improvement over stage 1 (DeLong test P<0.001; NRI = 0.630, IDI = 0.072; **Supplementary Figure 5A**). The SES ensemble model reached the optimal AUC of 0.731 (95% CI: 0.704-0.757) at stage 3, while the improvement was not statistically significant from stage 2. Notably, stage 3 still demonstrated a significant predictive advantage relative to the baseline stage (NRI = 0.470, IDI = 0.083, P < 0.001).

**Figure 2:**
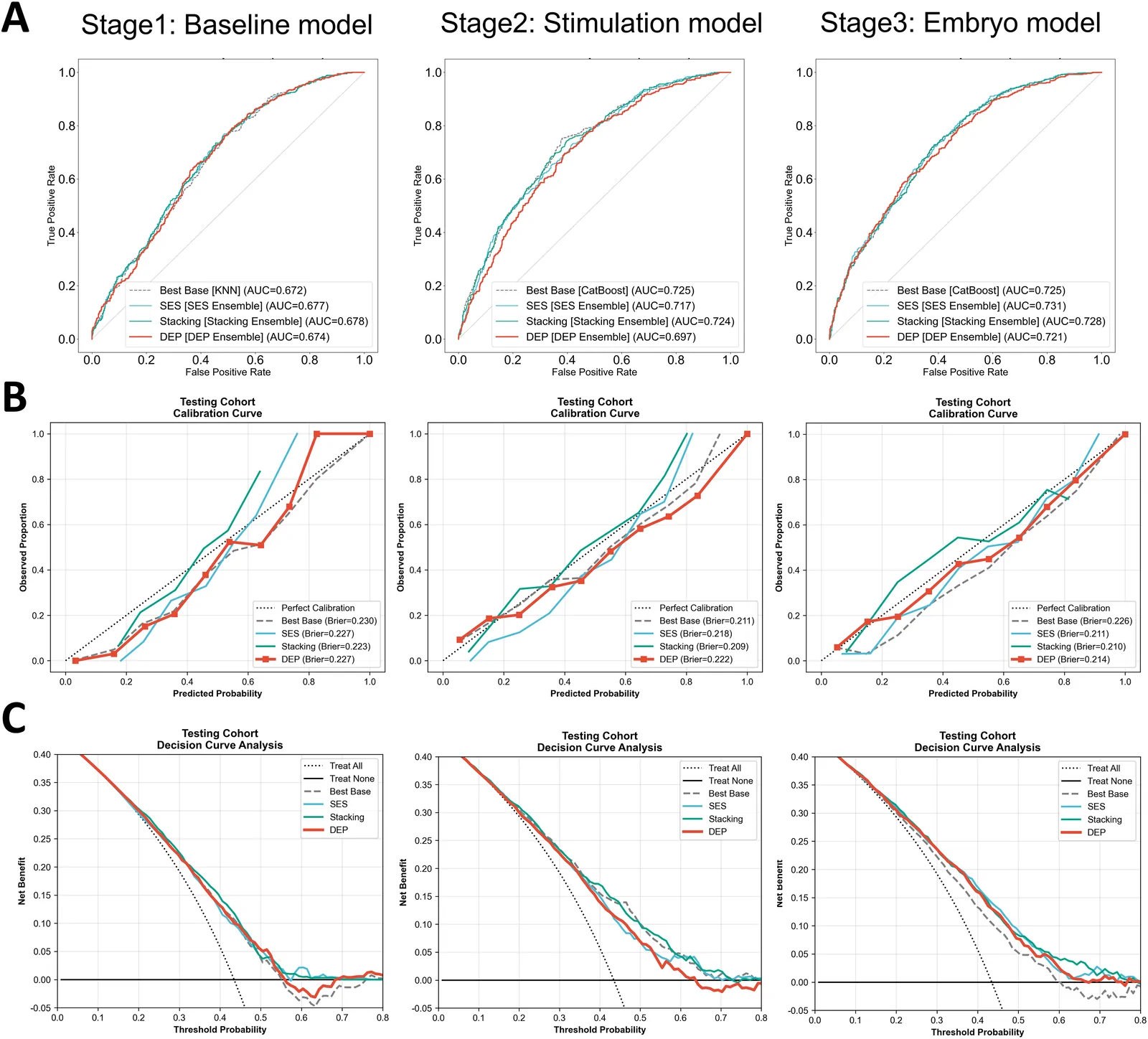
Performance comparison of the optimal base model and three ensemble strategies across three stages in test cohort. **(A)** ROC curves showing the discriminative performance at Stage 1, baseline stage; Stage 2, stimulation stage; and Stage 3 embryo development stages. **(B)** Calibration curves, with Brier scores noted. **(C)** Decision curve analysis.

**Table 2:** Performance of different machine learning models for predicting live birth outcome across three stages in test cohort.

Stage	Model	AUC (95% CI)	AUPRC (95% CI)	Brier Score	Accuracy	Sensitivity	Specificity	PPV	NPV	F1 score
1	Best Base	0.672 (0.643-0.702)	0.584 (0.543-0.628)	0.23	0.625	0.762	0.52	0.549	0.74	0.639
	SES	0.677 (0.649-0.707)	0.588 (0.547-0.633)	0.227	0.625	0.77	0.514	0.549	0.744	0.641
	Stacking	0.678 (0.649-0.708)	0.588 (0.547-0.633)	0.223	0.628	0.735	0.546	0.554	0.728	0.632
	DEP	0.674 (0.644-0.705)	0.591 (0.551-0.633)	0.227	0.619	0.79	0.487	0.542	0.751	0.643
2	Best Base	0.725 (0.698-0.751)	0.646 (0.604-0.690)	0.211	0.678	0.753	0.619	0.603	0.766	0.67
	SES	0.717 (0.687-0.742)	0.636 (0.595-0.679)	0.218	0.647	0.728	0.586	0.574	0.737	0.642
	Stacking	0.724 (0.695-0.749)	0.644 (0.603-0.689)	0.209	0.664	0.742	0.604	0.59	0.753	0.657
	DEP	0.697 (0.668-0.725)	0.611 (0.572-0.655)	0.222	0.647	0.697	0.61	0.578	0.723	0.632
3	Best Base	0.725 (0.698-0.753)	0.641 (0.601-0.686)	0.209	0.665	0.72	0.622	0.594	0.743	0.651
	SES	0.731 (0.704-0.757)	0.655 (0.616-0.700)	0.211	0.65	0.819	0.52	0.567	0.789	0.67
	Stacking	0.728 (0.701-0.755)	0.647 (0.608-0.691)	0.21	0.665	0.728	0.617	0.593	0.747	0.654
	DEP	0.721 (0.693-0.748)	0.653 (0.615-0.694)	0.214	0.666	0.616	0.704	0.615	0.705	0.616

Stage 1, baseline stage, the pre-IVF assessment period; Stage 2, stimulation stage, the ovarian stimulation phase; Stage 3, embryo development stage, embryological data including characteristics of embryos selected for transfer; AUC, area under the receiver operating characteristic curve; AUPRC, area under the precision-recall curve; PPV, positive predictive value; NPV, negative predictive value; SES, Static Ensemble Selection; DEP, Dynamic Ensemble Pruning; Best Base, best-performing base model.

PR-AUC increased sequentially from the baseline to the embryo development stage (**Supplementary Figure 5B**). Calibration curves showed good agreement between predicted and observed live birth rates (**Figure 2B**). DCA was conducted to quantify the clinical net benefit (**Figure 2C**). Probability-stratified live-birth rates based on prespecified clinical risk cutoffs are shown in **Supplementary Table 4**, illustrating observed live-birth outcomes across different predicted-risk subgroups.

### External validation of the predictive models

Model performance was further verified in an independent external validation cohort of 2,058 patients. Baseline characteristics by live birth outcome are presented in **Supplementary Table 5**. Patients with live birth were significantly younger, with higher AMH and AFC, lower BMI, and a lower rate of advanced age or DOR (all P < 0.05). **Supplementary Table 6** demonstrated women with live birth had lower gonadotropin dosage, higher peak E2, thicker endometrium, more oocytes retrieved, and higher blastocyst formation rates (all P < 0.001).

**Supplementary Table 7** summarizes the model performance in the final stages. External validation shows slightly lower discrimination than internal testing, due to inter-center differences in patient profiles and clinical practice. Among the models, the SES ensemble performed optimally and consistently at all stages, achieving the highest AUC of 0.719 (95% CI: 0.696-0.741) at the embryo development stage (**Figures 3A–C**). Calibration was acceptable across all models, with Brier scores ranging from 0.213 to 0.230. The findings supported favorable model generalizability.

**Figure 3:**
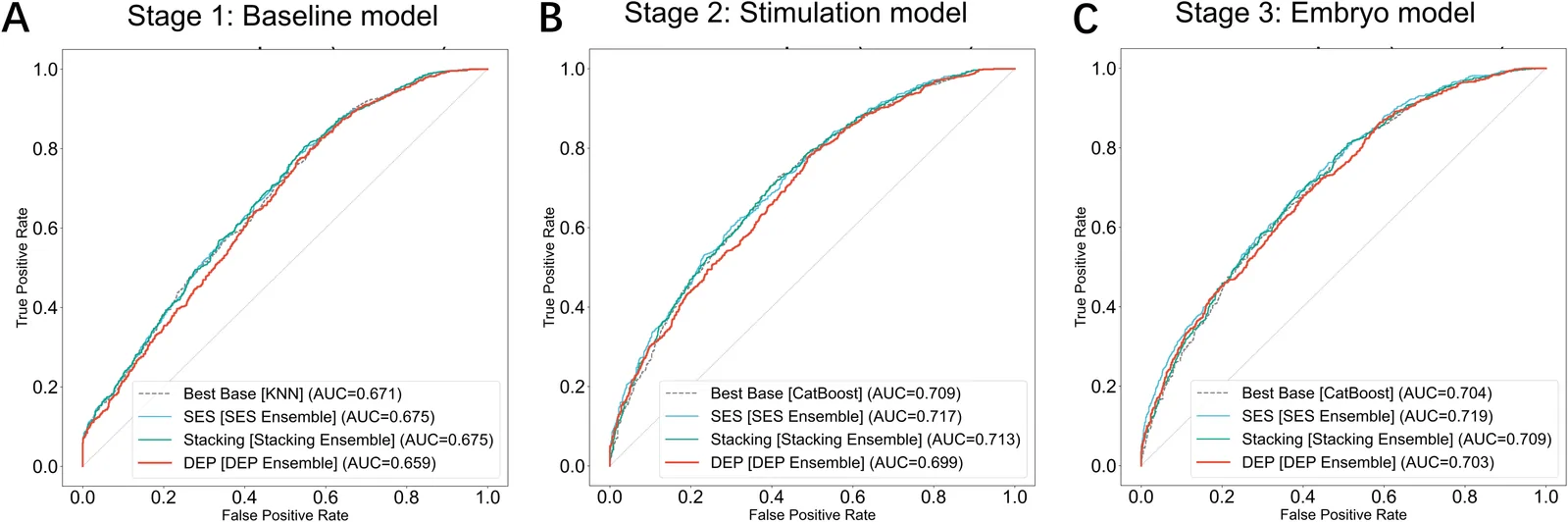
Performance of machine learning models across three stages in the external validation cohort. ROC curves showing the discriminative performance of three stages. **(A)** Stage 1, baseline stage, **(B)** Stage 2, stimulation stage, **(C)** Stage 3, embryo development stages.

### Model interpretability using SHAP analysis

To identify key predictors of live birth, we performed SHAP analysis across three sequential IVF/ICSI stages. Female age emerged as the most important predictor across all stages, exerting a strong negative impact on live birth likelihood. In the baseline stage (**Figure 4A**), AFC and male age were also key variables. In the stimulation stage (**Figure 4B**), down-regulation dose, trigger-day progesterone and EMT were the second important predictors. In the embryo development stage (**Figure 4C**), the number of high-quality embryos transferred and trigger-day progesterone were the next most influential predictors, followed by high-quality blastocysts and EMT. Bar plots showed the mean SHAP values for all features (**Supplementary Figures 6A–C**). These results confirmed the stepwise contributions of clinical, hormonal, and embryological data across the IVF/ICSI treatment pathway.

**Figure 4:**
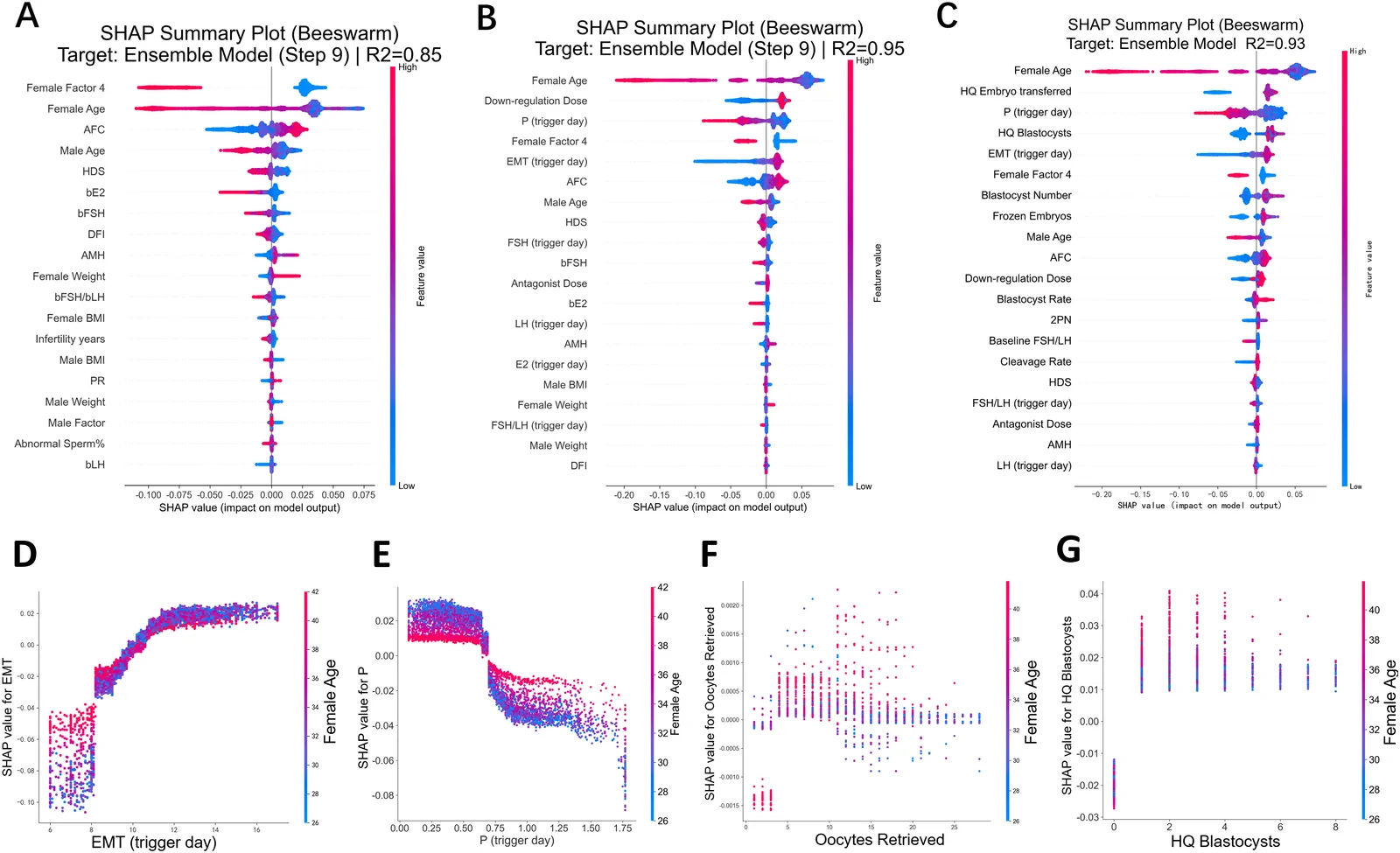
SHAP analysis revealing feature importance and dependence in the ensemble model across three stages. SHAP summary plot (beeswarm) showing the relative importance of variables in predicting live birth **(A)** baseline stage, **(B)** stimulation stage, **(C)** embryo development stage). **(D)** SHAP dependence plot showing the effect of trigger-day endometrial thickness (EMT) on model-predicted live birth probability, stratified by female age. **(E)** SHAP dependence plot depicting the association between trigger-day progesterone (P) and live birth prediction, stratified by female age. **(F)** SHAP dependence plot of the number of oocytes retrieved, demonstrating its impact on live birth prediction across different female age groups. **(G)** SHAP dependence plot of high-quality (HQ) blastocyst count, visualizing its contribution to live birth prediction by female age. Female factor 4 indicated advanced age/DOR.

SHAP dependence plots identified key effect modifiers. The negative impact of thin endometrium (<8 mm) was attenuated in older women, and the positive effect of EMT >10 mm was also less pronounced in this group (**Figure 4D**). Elevated trigger-day progesterone was more detrimental in younger women (**Figure 4E**). Oocyte yield and high-quality blastocysts conferred greater benefits to older women (**Figures 4F, G**). As shown in **Supplementary Figure 6D**, high AMH and AFC mitigated the adverse impact of advanced female age, while younger women were less affected by poor ovarian response. Down-regulation dose showed optimal outcomes at 1–3.75 mg. Critically, neither thin endometrium nor elevated progesterone could be rescued by increasing the number of transferred high-quality embryos.

### Nonlinear relationships by RCS analysis

RCS analysis revealed significant non-linear associations between key continuous variables and live birth probability (**Supplementary Figure 7**, **Supplementary Table 8**). Female and male age exhibited curvilinear trends. AMH and AFC were also non-linearly positively associated with live birth probability. Down-regulation and antagonist doses exhibited age-dependent non-linear effects. For women under 35, optimal range of down-regulation dose is approximately 2.5–3.0 mg. Trigger-day hormone profiles were also stratified by female age: in women aged ≥35, FSH and LH showed significant negative linear trends, whereas E2 followed an inverted U-shaped curve. Progesterone demonstrated a linear negative association in women <35 years. EMT on trigger day plateaued at 12mm, with no additional benefit beyond this threshold. Oocytes retrieved, cleaved embryos, high-quality blastocysts, and frozen embryos all showed threshold-dependent benefits, peaking at counts of 15, 11, 4, and 6 respectively before declining.

### Subgroup analysis

We further assessed model performance across clinically relevant subgroups to evaluate its robustness (**Supplementary Figure 8**). While the overall AUC was 0.730 (95% CI: 0.704–0.757), significant heterogeneity was detected in subgroups stratified by infertility factors, female age, oocytes retrieved, infertility type, and PCOS status (all P for interaction < 0.05). Specifically, the model performed better in patients with endometriosis (AUC = 0.799) or DOR (AUC = 0.787), secondary infertility (AUC = 0.753), age ≥35 years (AUC = 0.751), and ≥10 oocytes retrieved (AUC = 0.751). Poorer performance was seen in those with ovulatory disorder (AUC = 0.631), age <35 years (AUC = 0.671), PCOS (AUC = 0.628), and primary infertility (AUC = 0.687). No significant interactions were found for other factors.

## Discussion

This study developed and externally validated a three-stage sequential ensemble model for live-birth prediction in fresh IVF/ICSI cycles. Among ten base learners and three ensemble strategies, the SES ensemble demonstrated robust discrimination, calibration, and clinical net benefit across internal and external cohorts. The largest predictive improvement occurred after incorporating ovarian-stimulation-related variables at Stage 2, and SHAP analysis identified female age and transferred high-quality embryos as top predictive features.

Live birth after IVF/ICSI depends on sustained implantation, factors including embryo viability, euploidy, placental function, endometrial receptivity, immune and obstetric factors, representing a more clinically meaningful endpoint than clinical pregnancy (11, 12). Although many machine learning models predict clinical pregnancy (13, 14), fewer focus on live birth. Live birth is a complex multifactorial long-term outcome affected by many maternal variables not recorded in routine clinical data, inherently limiting predictive accuracy and resulting in moderate AUC values. Even so, our staged ensemble model achieved comparable discrimination to previously reported live birth prediction models (7, 15). Our three-stage framework mirrors real clinical decision-making: initial counseling based on baseline characteristics, mid-cycle decisions guided by ovarian response, and embryo transfer planning informed by embryological data. Adding stimulation parameters in Stage 2 significantly improved performance over Stage 1, reflecting the incremental value of ovarian response data. Adding embryological data in Stage 3 did not statistically improve prediction. One possible explanation is that ovarian response and endocrine profiles may already reflect key aspects of embryo competence (16). Another is that morphological grading is an imperfect surrogate for euploidy, such that high-quality embryos do not always result in live birth. Consequently, the limited AUC gain does not negate the clinical importance of embryological data, which remains valuable for post-transfer refinement rather than overall discrimination. Moreover, embryological features may yield individual-level risk re-stratification before embryo transfer within this modelling framework, though it was only observed in the test cohort and requires further external confirmation. Our three-stage framework is designed to deliver probability estimates at sequential clinical checkpoints, and Stage 3 corresponds specifically to the time point immediately prior to embryo transfer. Future integration of additional embryo-related biomarkers such as time-lapse morphokinetic parameters or preimplantation genetic testing for aneuploidy (PGT-A) may further boost predictive performance.

Our model incorporates a comprehensive panel of demographic, clinical, endocrine, and embryological variables, including male factors that are frequently underreported in other prediction models (17, 18). To minimize overfitting and ensure robust predictor selection, we applied a stringent multi-algorithm voting strategy combined with a clinical whitelist. Only variables supported by at least two algorithms or considered clinically indispensable were retained. This multi-modal selection approach differs from the single-method dimensionality-reduction strategies. Ensemble learning reduces prediction variance and improves stability by integrating complementary strengths of multiple base learners, thereby better capturing nonlinear biological interactions and enhancing external generalizability in IVF prediction (19). Compared with existing models, our study has notable strengths: a three-stage sequential design, two-center external validation, systematic ensemble optimization, and comprehensive interpretability analyses (SHAP and RCS). These features collectively enhance model transparency, generalizability, and clinical applicability. Among all ensemble strategies, SES yielded the highest AUC at the embryo development stage in both internal test and external validation cohorts while maintaining stable performance across all clinical stages. It also achieved the optimal PR-AUC and the lowest Brier score in external validation, indicating superior predictive precision and reliable probability calibration. SES also exhibits favorable feasibility for future translation given its relatively concise ensemble structure.

SHAP analysis identified female age ranked highest across all stages, consistent with previous studies (20, 21). At baseline, male factors including male age, sperm DFI and HDS also showed strong predictive importance, supporting the value of joint couple assessment before treatment (22–24). In the stimulation phase, down-regulation dose, trigger-day progesterone and EMT were key predictors, consistent with known drivers of implantation (25, 26). RCS revealed the nonlinear relationships: EMT reached a plateau at 12 mm, with no additional benefit beyond this threshold. In the embryo development stage, number of transferred high-quality embryos and total high-quality blastocysts dominated prediction, confirming embryo quality as a strong predictor of live birth (27). SHAP dependence plots also reflected age-related heterogeneity: elevated progesterone exerted stronger adverse impacts on younger women, while higher EMT provided less benefit in older patients. Sufficient oocyte yield and good blastocyst quality benefited older women more, and intact ovarian reserve could partly offset the negative impact of advanced age. A key clinical implication is that poor endometrial condition or elevated progesterone cannot be compensated by transferring more high-quality embryos. Moderate oocyte and blastocyst numbers were sufficient for optimal outcomes, supporting a quality-over-quantity approach in clinical stimulation (28). Subgroup analysis defined the model’s applicable population, showing better performance in patients with advanced age, endometriosis and DOR, and relatively lower efficacy in those with PCOS.

This study had several limitations. First, its retrospective design carried inherent risks of selection bias and unmeasured confounding. Although we used data from two independent centers, prospective and multi-center studies are needed to improve generalizability, its performance in other ethnic groups requires further evaluation. Clinically actionable probability thresholds for individual-patient management remain to be validated in prospective cohorts. Second, preimplantation genetic testing and frozen embryo transfer cycles were excluded, limiting applicability to these settings. Third, lifestyle, psychological factors, and reproductive immune indicators were not available and could not be included. Fourth, we only adopted binary live birth outcome as the endpoint, without distinguishing biochemical pregnancy, early miscarriage and clinical pregnancy as separate predictive endpoints. Fifth, obstetric factors affecting ongoing pregnancy were not included, and integrating such data may further improve predictive performance.

## Conclusions

This validated three-stage prediction model enables dynamic, stage-specific live birth assessment in fresh IVF/ICSI cycles that proceed to embryo transfer. Female age and number of transferred high-quality embryos were the top-ranked predictors in our model, with embryo quality showing greater predictive weight for live-birth probability than oocyte or blastocyst yield. This work may inform personalized patient counselling, though prospective validation of clinical decision thresholds is required before real-world clinical application.

## Data Availability

The raw data supporting the conclusions of this article will be made available by the authors, without undue reservation.
