# Supplementary material for: Development and external validation of a three-stage model to predict live birth after fresh IVF/ICSI embryo transfer
Source: Front Endocrinol (Lausanne). 2026 Sep 21;17:1916182. doi: 10.3389/fendo.2026.1916182 (PMC13635321; doi:10.3389/fendo.2026.1916182)
Supplement: Supplementary file 1 [file SupplementaryFile1.docx]

**SUPPLEMENTARY FIGURES AND TABLES**

**List of Supplementary Materials:**

**Figure S1** Feature selection across three sequential IVF/ICSI stages.

A, D, G: LASSO mean squared error (MSE) path showing the optimal penalty parameter (A. baseline stage, D. stimulation stage, G. embryo stage); B, E, H: LASSO coefficient shrinkage path (B. baseline stage, E. stimulation stage, H. embryo stage); C, F, I: Random Forest Gini importance plot, ranking features by their predictive value for live birth (C. baseline stage, F. stimulation stage, I. embryo stage).

**Figure S2** Algorithm intersection network showing consensus features selected by at least two of the four methods (LASSO, Random Forest, RFE, and Boruta) across three stages. A. baseline stage; B. stimulation stage; C. embryo stage. RF: Random Forest, RFE: Recursive Feature Elimination.

**Figure S3** Performance of 10 base machine learning models across three stages in the internal validation cohort. A. ROC curves showing the discriminative performance at Stage 1 (baseline), Stage 2 (stimulation), and Stage 3 (embryo) stages. B. Precision-recall curves. C. Calibration curves, with Brier scores noted. D. Decision curve analysis.

**Figure S4** Performance comparison of the optimal base model and three ensemble strategies across three stages in the internal validation cohort. A. ROC curves showing the discriminative performance at Stage 1 (baseline), Stage 2 (stimulation), and Stage 3 (embryo) stages. B. Precision-recall curves. C. Calibration curves, with Brier scores noted. D. Decision curve analysis.

**Figure S5** Model performance improvement and testing cohort evaluation across three stages. A. Stepwise improvement in testing AUC from baseline to stimulation and embryo transfer, with DeLong test P-values, net reclassification improvement (NRI), and integrated discrimination improvement (IDI) indicated. B. Precision-recall curves for the base and ensemble models at each stage in the testing cohort, with AUPRC values noted.

**Figure S6** SHAP analysis of feature importance and dependence in the ensemble model. Bar plots showing mean absolute SHAP values for all features, ranked by importance (A. baseline stage, B. stimulation stage, C. embryo stage). D. SHAP dependence plots illustrating the relationship between selected feature values and their corresponding SHAP values, colored by interacting variables.

**Figure S7** Restricted cubic spline (RCS) curves showing the non-linear relationships between key variables and the live birth probability. Variables include female and male age, ovarian reserve markers (AMH, AFC), stimulation parameters (cos days, down-regulation dose, antagonist dose, trigger-day hormones and endometrial thickness), and embryological outcomes (oocytes retrieved, blastocyst development, cleavage rate). P for overall: significance of the overall association; P for non-linear: significance of the nonlinear spline terms.

**Figure S8** Forest plot of subgroup analysis showing model AUC performance across clinical subgroups. The plot displays the AUC (with 95% confidence intervals) for the overall cohort and subgroups defined by female factors, male factors, age, BMI, AMH, oocyte count, infertility type, and PCOS status. Female factor 1-5: tubal/peritoneal, endometriosis, ovulatory disorder, advanced age/DOR, unexplained. *P* values for interaction testing.

**Supplementary table 1**. Ovarian stimulation, oocyte retrieval, and embryo development characteristics of the study population across four cohorts

| Factors | Overall (n=10447) | Training Cohort | | | Internal validation (n=1259) | P | Testing cohort (n=1259) | P | External validation (n=2058) | P |
| --- | --- | --- | --- | --- | --- | --- | --- | --- | --- | --- |
|  |  | Non-Live Birth (n=3386) | Live birth (n=2485) | P |  |  |  |  |  |  |
| **COS Protocol**, n (%) | |  |  | **<0.001** |  | 0.801 |  | 0.173 |  | 0.083 |
| Antagonist protocol | 3852 (36.9) | 1383 (40.8) | 785 (31.6) |  | 454 (36.1) |  | 431 (34.2) |  | 799 (38.8) |  |
| Long protocol | 6044 (57.9) | 1776 (52.5) | 1629 (65.6) |  | 743 (59.0) |  | 757 (60.1) |  | 1139 (55.3) |  |
| Total Gn dose, IU | 2475 [1800, 3075] | 2550 [1950, 3150] | 2475 [1875, 3150] | 0.157 | 2479 [1950, 3025] | 0.803 | 2475 [1950, 3223] | 0.871 | 2250 [1606, 3000] | **<0.001** |
| Starting Gn dose, IU | 225 [187, 300] | 225 [225, 300] | 225 [187, 300] | **<0.001** | 225 [200, 300] | 0.357 | 225 [200, 300] | 0.171 | 225 [150, 300] | **<0.001** |
| COS days | 10 [9, 12] | 10[9, 11] | 11 [10, 12] | **<0.001** | 10 [9, 11] | 0.328 | 10 [9, 12] | 1.000 | 11 [9, 12] | **<0.001** |
| Down-regulation Dose | 3.75 [1.88, 3.75] | 3.75 [1.20, 3.75] | 3.75 [1.35, 3.75] | **0.013** | 3.75 [1.25, 3.75] | 0.534 | 3.75 [1.25, 3.75] | 0.913 | 1.30 [1.00, 3.75] | **<0.001** |
| Antagonist Dose | 1.25 [1.00, 1.50] | 1.25 [1.00, 1.50] | 1.25 [1.00, 1.50] | 0.069 | 1.25 [1.00, 1.50] | 0.670 | 1.25 [1.00, 1.50] | 0.926 | 1.25 [1.00, 1.50] | **<0.001** |
| **Trigger Day Hormones** | |  |  |  |  |  |  |  |  |  |
| FSH, IU/L | 16.00 [12.60, 19.60] | 16.24 [12.90, 19.80] | 15.70 [12.40, 19.16] | **<0.001** | 16.28 [12.90, 19.89] | 0.151 | 16.49 [12.98, 19.91] | 0.094 | 15.12 [11.80, 19.00] | **<0.001** |
| LH, IU/L | 1.73 [1.12, 2.88] | 1.89 [1.21, 3.33] | 1.76 [1.18, 2.77] | **<0.001** | 1.79 [1.20, 2.92] | 0.345 | 1.79 [1.19, 2.82] | 0.113 | 1.37 [0.81, 2.32] | **<0.001** |
| FSH/LH ratio | 8.61 [4.85, 13.48] | 8.33 [4.53, 13.12] | 8.82 [5.15, 13.48] | **0.001** | 8.61 [4.90, 13.75] | 0.604 | 8.86 [5.25, 13.54] | 0.132 | 8.53 [4.74, 13.87] | 0.541 |
| E2, pg/ml | 1998 [1279, 2927] | 1843 [1162, 2833] | 2064 [1366, 2962] | **<0.001** | 1929.00 [1268.50, 2905.00] | 0.858 | 1953.59 [1272.75, 2900.50] | 0.632 | 2181.00 [1422.62, 3119.89] | **<0.001** |
| P, ng/ml | 0.59 [0.37, 0.86] | 0.62 [0.40, 0.89] | 0.56 [0.36, 0.83] | 0.002 | 0.59 [0.36, 0.86] | 0.792 | 0.58 [0.37, 0.86] | 0.778 | 0.60 [0.40, 0.86] | **0.002** |
| Endometrial thickness, mm | 10.90 [9.50, 12.40] | 10.70 [9.20, 12.30] | 11.20 [9.90, 12.80] | **<0.001** | 10.90 [9.50, 12.40] | 0.421 | 11.00 [9.60, 12.45] | 0.593 | 10.60 [9.10, 12.00] | **<0.001** |
| **Embryology Data** | |  |  |  |  |  |  |  |  |  |
| Oocytes Retrieved | 10 [6, 14] | 8 [5, 14] | 11 [7, 15] | **<0.001** | 10 [6, 15] | 0.796 | 10 [6, 15] | 0.629 | 10 [6, 14] | **0.016** |
| MII Oocytes | 8 [5, 12] | 7 [4, 12] | 9 [6, 13] | **<0.001** | 9 [5, 13] | 0.898 | 9 [5, 12] | 0.754 | 8 [5, 12] | 0.207 |
| ICSI, n (%) | 1904 (18.2) | 721 (21.3) | 399 (16.1) | **<0.001** | 222 (17.6) | 0.486 | 228 (18.1) | 0.573 | 334 (16.2) | **0.014** |
| 2PN | 5 [3, 9] | 5 [3, 8] | 6 [4, 9] | **<0.001** | 5 [3, 9] | 0.825 | 5 [3, 9] | 0.743 | 6 [3, 8] | 0.643 |
| Cleaved Embryos | 6 [3, 10] | 6 [3, 9] | 7 [4, 10] | **<0.001** | 6 [3, 10] | 0.493 | 6 [3, 10] | 0.967 | 7 [4, 10] | **<0.001** |
| Frozen Embryos | 2 [0, 4] | 1 [0, 3] | 2 [1, 4] | **<0.001** | 2 [0, 4] | 0.695 | 2 [0, 4] | 0.557 | 2 [0, 4] | 0.739 |
| Blastocyst formed | 3 [1, 6] | 3 [1, 6] | 4 [2, 7] | **<0.001** | 4 [1, 6] | 0.825 | 4 [2, 6] | 0.760 | 2 [0, 5] | **<0.001** |
| Blastocyst Rate | 0.58 [0.33, 0.77] | 0.55 [0.33, 0.75] | 0.64 [0.43, 0.80] | **<0.001** | 0.60 [0.34, 0.78] | 0.766 | 0.60 [0.38, 0.77] | 0.990 | 0.50 [0.31, 0.71] | **<0.001** |
| Embryo transfer days, n (%) | | |  | **<0.001** |  | 0.677 |  | **0.032** |  | **<0.001** |
| D3 | 6503 (62.2) | 2147 (63.4) | 1369 (55.1) |  | 759 (60.3) |  | 749 (59.5) |  | 1479 (71.9) |  |
| D5 | 3707 (35.5) | 1156 (34.1) | 1077 (43.3) |  | 470 (37.3) |  | 478 (38.0) |  | 526 (25.6) |  |
| Transfer number, n (%) | |  |  | 0.064 |  | 0.631 |  | 0.625 |  | **<0.001** |
| 1 | 4513 (43.2) | 1441 (42.6) | 1119 (45.0) |  | 559 (44.4) |  | 539 (42.8) |  | 855 (41.5) |  |
| 2 | 5923 (56.7) | 1944 (57.4) | 1366 (55.0) |  | 700 (55.6) |  | 720 (57.2) |  | 1193 (58.0) |  |
| High-quality Embryos transferred, n (%) | | | | **<0.001** |  | 0.517 |  | 0.408 |  | **<0.001** |
| 0 | 2396 (22.9) | 966 (28.5) | 421 (16.9) |  | 284 (22.6) |  | 319 (25.3) |  | 406 (19.7) |  |
| 1 | 5749 (55.0) | 1736 (51.3) | 1546 (62.2) |  | 727 (57.7) |  | 683 (54.2) |  | 1057 (51.4) |  |
| 2 | 2276 (21.8) | 677 (20.0) | 511 (20.6) |  | 247 (19.6) |  | 253 (20.1) |  | 588 (28.6) |  |

Data are presented as median [interquartile range] for continuous variables and n (%) for categorical variables. *Abbreviations: COS, Controlled Ovarian Stimulation; Gn, gonadotropin; LH, luteinizing hormone; HCG, human chorionic gonadotropin; E2, estradiol; FSH, follicle-stimulating hormone; P, Progesterone; ICSI, intracytoplasmic sperm injection; D3, day 3; D5, day 5; 2PN, Two Pronuclei. P-value: Comparison across training, internal validation, testing, and external validation cohorts. P-value†: Comparison between live birth and non-live birth groups in the overall cohort.

**Supplementary table 2. Results of Feature Selection by Multiple Algorithms**

| Feature | Lasso Selected | Lasso Score (Absolute Coefficient) | RF Selected | RF Score (Gini) | RFE Selected | Boruta Selected | Total Votes | Final Decision |
| --- | --- | --- | --- | --- | --- | --- | --- | --- |
| Female Age | Yes | 0.0840 | Yes | 0.0469 | Yes | Yes | 5 | Protected (Whitelist) |
| P (trigger day) | Yes | 0.0359 | Yes | 0.0321 | Yes | Yes | 5 | Retained |
| EMT (trigger day) | Yes | 0.0256 | Yes | 0.0316 | Yes | Yes | 5 | Retained |
| Male Age | Yes | 0.0064 | Yes | 0.0306 | Yes | Yes | 5 | Protected (Whitelist) |
| AMH | Yes | 0.0155 | Yes | 0.0300 | Yes | Yes | 5 | Protected (Whitelist) |
| HDS | Yes | 0.0104 | Yes | 0.0299 | Yes | Yes | 5 | Retained |
| E2 (trigger day) | Yes | 0.0047 | Yes | 0.0294 | Yes | Yes | 5 | Retained |
| Female Weight | Yes | 0.0079 | Yes | 0.0290 | Yes | Yes | 5 | Retained |
| AFC | Yes | 0.0208 | Yes | 0.0224 | Yes | Yes | 5 | Retained |
| Blastocyst number | No | 0 | Yes | 0.0218 | Yes | Yes | 4 | Retained |
| Down-regulation Dose | Yes | 0.0026 | Yes | 0.0213 | No | Yes | 4 | Retained |
| bFSH | Yes | 0.0088 | Yes | 0.0289 | Yes | No | 3 | Protected (Whitelist) |
| FSH (trigger day) | Yes | 0.0016 | Yes | 0.0275 | Yes | No | 3 | Protected (Whitelist) |
| Male BMI | Yes | 0.0049 | Yes | 0.0274 | Yes | No | 3 | Protected (Whitelist) |
| PR | Yes | 0.0065 | Yes | 0.0274 | Yes | No | 3 | Retained |
| FSH/LH (trigger day) | Yes | 0.0143 | Yes | 0.0273 | Yes | No | 3 | Protected (Whitelist) |
| bLH | Yes | 0.0020 | Yes | 0.0271 | Yes | No | 3 | Retained |
| bE2 | Yes | 0.0114 | Yes | 0.0270 | Yes | No | 3 | Protected (Whitelist) |
| Female BMI | Yes | 0.0101 | Yes | 0.0270 | Yes | No | 3 | Protected (Whitelist) |
| Blastocyst Rate | Yes | 0.0186 | Yes | 0.0239 | Yes | No | 3 | Retained |
| Antagonist Dose | Yes | 0.0099 | Yes | 0.0238 | Yes | No | 3 | Retained |
| Frozen Embryos | Yes | 0.0042 | No | 0.0156 | No | Yes | 3 | Retained |
| Female Factor 4 | Yes | 0.0292 | No | 0.0139 | No | Yes | 3 | Retained |
| HQ Embryos transferred | Yes | 0.0536 | No | 0.0128 | No | Yes | 3 | Retained |
| HQ Blastocysts | Yes | 0.0069 | No | 0.0127 | No | Yes | 3 | Protected (Whitelist) |
| DFI | No | 0 | Yes | 0.0290 | Yes | No | 2 | Retained |
| E2 per Oocytes | No | 0 | Yes | 0.0289 | Yes | No | 2 | Retained |
| Male Weight | No | 0 | Yes | 0.0283 | Yes | No | 2 | Retained |
| bFSH/bLH | No | 0 | Yes | 0.0278 | Yes | No | 2 | Protected (Whitelist) |
| LH (trigger day) | No | 0 | Yes | 0.0270 | Yes | No | 2 | Retained |
| Abnormal Sperm% | No | 0 | Yes | 0.0237 | Yes | No | 2 | Retained |
| Total Gn | Yes | 0.0062 | Yes | 0.0210 | No | No | 2 | Retained |
| Oocytes Retrieved | Yes | 0.0131 | Yes | 0.0205 | No | No | 2 | Protected (Whitelist) |
| Cleavage Rate | Yes | 0.0160 | Yes | 0.0199 | No | No | 2 | Retained |
| MII Rate | Yes | 0.0038 | No | 0.0184 | No | No | 1 | Dropped |
| 2PN | Yes | 0.0154 | No | 0.0181 | No | No | 1 | Dropped |
| COS days | Yes | 0.0083 | No | 0.0132 | No | No | 1 | Dropped |
| Starting Gn | Yes | 0.0017 | No | 0.0092 | No | No | 1 | Dropped |
| COS Protocol | Yes | 0.0399 | No | 0.0049 | No | No | 1 | Dropped |
| Fertilization | Yes | 0.0227 | No | 0.0045 | No | No | 1 | Dropped |
| Embryo transfer days | Yes | 0.0369 | No | 0.0041 | No | No | 1 | Dropped |
| Transfer number | Yes | 0.0230 | No | 0.0041 | No | No | 1 | Dropped |
| LH Added | Yes | 0.0081 | No | 0.0037 | No | No | 1 | Dropped |
| Infertility years | No | 0 | No | 0.0171 | No | No | 0 | Protected (Whitelist) |
| Male Factor | No | 0 | No | 0.0034 | No | No | 0 | Protected (Whitelist) |

Notes: AFC, antral follicle count; AMH, anti-Müllerian hormone; bE2, basal estradiol; bFSH, basal follicle-stimulating hormone; bLH, basal luteinizing hormone; COS, controlled ovarian stimulation; DFI, DNA fragmentation index; EMT, endometrial thickness; FSH, follicle-stimulating hormone; Gn, gonadotropin; HDS, high DNA stainability; HQ, high-quality; LH, luteinizing hormone; P, progesterone; PR, progressive motility; Female Factor 4: Advanced age/Diminished ovarian reserve; RFE, recursive feature elimination; RF, random forest. Total Votes counts positive selections from four algorithms (Lasso, RF, RFE, Boruta; range 0‑4). Clinically‑important variables form an independent pre‑defined clinical whitelist rule and are not counted within algorithm‑derived votes. Final Decision: Protected (Whitelist): retained regardless of the number of votes; Retained = features selected by ≥2 algorithms; Dropped = features selected by <2 algorithms and without clinical priority.

**Supplementary Table 3. Selection results of Key Variables in Three Stages of IVF/ICSI Cycle**

| IVF/ICSI Stage | Key Variables |
| --- | --- |
| Baseline Stage | Female/male age, Infertility years, Female/male BMI/weight, Female factor 4 (advanced age/DOR), Male factor, Baseline hormones (FSH, LH, E2, FSH/LH ratio), AMH, AFC, PR, DFI, HDS, Sperm abnormal rate |
| Ovarian Stimulation Stage | Baseline variables, Trigger Day hormones (E2, LH, FSH, P, FSH/LH ratio), Endometrial thickness, Down-regulation dose, Antagonist dose, Total Gn dose |
| Embryo Transfer Stage | Baseline variables, Ovarian stimulation variables, Cleavage rate, Blastocyst formation rate, Number of oocytes retrieved, blastocysts, high-quality blastocysts/transferred, embryos transferred, frozen embryos. |

AMH, anti-Müllerian hormone; AFC, antral follicle count; BMI, body mass index; DFI, DNA fragmentation index; DOR, diminished ovarian reserve; E2, estradiol; FSH, follicle-stimulating hormone; Gn, gonadotropin; HDS, high DNA stainability; LH, luteinizing hormone; P, progesterone; PR, progressive motility.

**Supplementary Table 4. Probability‑stratified observed live birth rates based on pre‑specified clinical risk cut‑offs**

| Probability Group | N | Live Birth | Live Birth Rate | Clinical Net Benefit | Decision Threshold | Clinical Recommendation |
| --- | --- | --- | --- | --- | --- | --- |
| Low (< 0.20) | 96 | 3 | 3.12% | 0.374 | 0.1 | Low predicted live‑birth probability |
| Intermediate-low (0.20–0.45) | 374 | 97 | 25.94% | 0.235 | 0.3 | Individualized counseling, pre‑transfer optimization |
| Intermediate-high (0.45–0.60) | 337 | 165 | 48.96% | 0.091 | 0.5 | Moderate, standard clinical protocols and monitoring |
| High (≥ 0.60) | 452 | 282 | 62.39% | 0.002 | 0.8 | Favorable, discussion of single‑embryo transfer policy |

**Supplementary Table 5. Baseline demographic and clinical characteristics of the study population in external validation cohort.**

| Characteristics | Overall (n=2058) | Non-live birth (n=1138) | Live birth (n=920) | P |
| --- | --- | --- | --- | --- |
| **Demographics** |  |  |  |  |
| Female Age, years | 32 [29, 36] | 33 [30, 37] | 31 [29, 34] | **<0.001** |
| Male Age, years | 35 [32, 38] | 35 [32, 39] | 34 [31, 37] | **<0.001** |
| Female BMI | 21.44 [19.98, 23.05] | 21.60 [20.06, 23.18] | 21.26 [19.81, 22.80] | **0.001** |
| Female Weight | 53.45 [50.39, 57.00] | 53.64 [50.40, 57.20] | 53.25 [50.22, 56.80] | 0.283 |
| Infertility Type [n (%)] |  |  |  | **<0.001** |
| Primary infertility | 903 (43.9) | 461 (40.5) | 442 (48.0) |  |
| Secondary infertility | 1155 (56.1) | 677 (59.5) | 478 (52.0) |  |
| **Infertility Causes, n (%)** | |  |  |  |
| Tubal/peritoneal factor | 1337 (65.0) | 736 (64.7) | 601 (65.3) | 0.794 |
| Endometriosis | 188 (9.1) | 104 (9.1) | 84 (9.1) | 1.000 |
| Ovulatory disorder | 364 (17.7) | 175 (15.4) | 189 (20.5) | **0.003** |
| Advanced age/DOR | 442 (21.5) | 329 (28.9) | 113 (12.3) | **<0.001** |
| Unexplained | 236 (11.5) | 126 (11.1) | 110 (12.0) | 0.578 |
| Male Factor | 874 (42.5) | 481 (42.3) | 393 (42.7) | 0.867 |
| **Baseline Hormones** |  |  |  |  |
| FSH, IU/L | 6.30 [5.06, 7.70] | 6.41 [5.18, 7.96] | 6.10 [4.95, 7.54] | **0.002** |
| LH, IU/L | 3.77 [2.68, 5.25] | 3.73 [2.59, 5.20] | 3.85 [2.74, 5.34] | 0.272 |
| FSH/LH | 1.66 [1.23, 2.28] | 1.72 [1.28, 2.41] | 1.60 [1.17, 2.14] | **<0.001** |
| E2, pg/mL | 36.46 [30.00, 46.96] | 37.00 [30.00, 47.53] | 36.00 [30.40, 46.00] | 0.416 |
| PRL, ng/ml | 15.26 [11.04, 21.56] | 14.85 [10.71, 21.17] | 15.85 [11.41, 22.31] | **0.010** |
| T, ng/ml | 0.29 [0.19, 0.39] | 0.29 [0.19, 0.40] | 0.28 [0.19, 0.39] | 0.966 |
| AMH, ng/mL | 2.70 [1.57, 4.28] | 2.42 [1.36, 4.06] | 2.96 [1.87, 4.57] | **<0.001** |
| AFC, n | 13 [8, 19] | 12 [8, 18] | 14 [9, 20] | **<0.001** |
| **Sperm Parameters** |  |  |  |  |
| Progressive motility, % | 47.00 [37.00, 56.00] | 46.60 [37.00, 55.40] | 47.75 [36.32, 56.25] | 0.448 |
| Concentration,  10⁶/mL | 53.29 [35.92, 75.65] | 53.59 [36.44, 76.78] | 52.49 [35.55, 73.30] | 0.454 |
| Abnormal Sperm, % | 97.00 [95.20, 98.20] | 97.00 [95.03, 98.06] | 97.00 [95.38, 98.20] | 0.673 |
| DFI, % | 19.09 [15.18, 24.29] | 19.27 [15.57, 24.44] | 18.65 [14.81, 24.01] | **0.042** |
| HDS, % | 8.55 [6.83, 10.76] | 8.48 [6.78, 10.68] | 8.63 [6.87, 10.77] | 0.416 |

Data are presented as median [interquartile range] for continuous variables and n (%) for categorical variables. †P-value comparing live birth and non-live birth groups in the external validation cohort. *Abbreviations: DOR, diminished ovarian reserve; BMI, body mass index; FSH, follicle-stimulating hormone; LH, luteinizing hormone; E2, estradiol; PRL, prolactin; AMH, anti-Müllerian hormone; AFC, antral follicle count; DFI, DNA fragmentation index. HDS, high DNA stainability

**Supplementary Table 6. Ovarian stimulation, oocyte retrieval, and embryo development characteristics of the study population in external validation cohort.**

| Characteristics | Overall (n=2058) | Non-live birth (n=1138) | Live birth (n=920) | P |
| --- | --- | --- | --- | --- |
| **COS Protocol**, n (%) |  |  |  | **<0.001** |
| Antagonist protocol | 799 (38.8) | 484 (42.5) | 315 (34.2) |  |
| Long protocol | 1139 (55.3) | 566 (49.7) | 573 (62.3) |  |
| Down-regulation Dose | 1.30 [1.00, 3.75] | 1.30 [1.00, 3.75] | 1.30 [1.00, 3.75] | 0.634 |
| Antagonist Dose | 1.25 [1.00, 1.50] | 1.25 [1.00, 1.50] | 1.25 [1.07, 1.50] | 0.255 |
| **Trigger Day Hormones** | |  |  |  |
| FSH, IU/L | 15.12 [11.80, 19.00] | 14.90 [11.61, 19.21] | 15.39 [12.00, 18.50] | 0.959 |
| LH, IU/L | 1.37 [0.81, 2.32] | 1.38 [0.81, 2.41] | 1.35 [0.81, 2.20] | 0.201 |
| FSH/LH ratio | 8.53 [4.74, 13.87] | 8.34 [4.55, 14.17] | 8.77 [5.18, 13.35] | 0.490 |
| E2, pg/ml | 2181.00 [1422.62, 3119.89] | 2067.57 [1296.46, 3038.15] | 2339.71 [1548.70, 3220.64] | **<0.001** |
| P, ng/ml | 0.60 [0.40, 0.86] | 0.61 [0.40, 0.89] | 0.60 [0.40, 0.83] | 0.153 |
| Endometrial thickness, mm | 10.60 [9.10, 12.00] | 10.40 [9.00, 12.00] | 10.90 [9.30, 12.10] | **<0.001** |
| **Embryology Data** |  |  |  |  |
| Oocytes Retrieved | 10 [6, 14] | 9 [5, 13] | 11 [7, 14] | **<0.001** |
| E2 per Oocytes | 227.44 [167.93, 314.52] | 232.00 [169.76, 321.55] | 221.62 [166.52, 304.78] | 0.054 |
| MII Oocytes | 8 [5, 12] | 8 [5, 11] | 9 [6, 12] | **<0.001** |
| MII Rate | 0.92 [0.80, 1.00] | 0.92 [0.80, 1.00] | 0.92 [0.81, 1.00] | 0.341 |
| Fertilization [n (%)] |  |  |  | **0.037** |
| ICSI | 380 (18.4) | 231 (20.3) | 149 (16.2) |  |
| IVF | 1678 (81.5) | 907 (79.7) | 771 (83.8) |  |
| 2PN | 6 [3, 8] | 5.00 [3, 8] | 6 [4, 9] | **<0.001** |
| Cleaved Embryos | 7 [4, 10] | 6 [3, 10] | 7 [4, 11] | **<0.001** |
| Frozen Embryos | 2 [0, 4] | 1 [0, 3] | 2 [1, 4] | **<0.001** |
| Blastocyst number | 2 [0, 5] | 2 [0, 4] | 2 [0, 5] | **<0.001** |
| Transfer number, n (%) | |  |  | **0.019** |
| 1 | 855 (41.5) | 504 (44.3) | 351 (38.2) |  |
| 2 | 1193 (58.0) | 629 (55.3) | 564 (61.3) |  |
| Embryo transfer days, n (%) | |  |  | 0.133 |
| D3 | 1479 (71.9) | 838 (73.6) | 641 (69.7) |  |
| D5 | 526 (25.6) | 270 (23.7) | 256 (27.8) |  |
| High-quality Embryos transferred, n (%) | |  |  | **<0.001** |
| 0 | 406 (19.7) | 277 (24.3) | 129 (14.0) |  |
| 1 | 1057 (51.4) | 569 (50.0) | 488 (53.0) |  |
| 2 | 595 (28.9) | 292 (25.7) | 303 (32.9) |  |

Data are presented as median [interquartile range] for continuous variables and n (%) for categorical variables. *Abbreviations: COS, Controlled Ovarian Stimulation; Gn, gonadotropin; LH, luteinizing hormone; E2, estradiol; FSH, follicle-stimulating hormone; P, Progesterone; IVF, In vitro Fertilization; ICSI, intracytoplasmic sperm injection; D3, day 3; D5, day 5; 2PN, Two Pronuclei. P-value†: Comparison between live birth and non-live birth groups in the external validation cohort.

**Supplementary Table 7. Performance of Machine Learning Models for Predicting Live Birth Outcome at the embryo development stage in External Validation**

| Model | AUC (95% CI) | AUPRC (95% CI) | Brier Score | Accuracy | Sensitivity | Specificity | PPV | NPV | F1 Score |
| --- | --- | --- | --- | --- | --- | --- | --- | --- | --- |
| Best Base | 0.704 (0.682-0.726) | 0.640 (0.609-0.674) | 0.22 | 0.632 | 0.796 | 0.499 | 0.562 | 0.751 | 0.659 |
| SES | 0.719 (0.696-0.741) | 0.678 (0.648-0.710) | 0.215 | 0.648 | 0.688 | 0.616 | 0.592 | 0.71 | 0.636 |
| Stacking | 0.709 (0.688-0.731) | 0.653 (0.622-0.687) | 0.215 | 0.634 | 0.811 | 0.49 | 0.563 | 0.762 | 0.664 |
| DEP | 0.703 (0.680-0.724) | 0.656 (0.626-0.689) | 0.22 | 0.635 | 0.68 | 0.598 | 0.578 | 0.698 | 0.625 |

Abbreviations: AUC, area under the receiver operating characteristic curve; AUPRC, area under the precision-recall curve; PPV, positive predictive value; NPV, negative predictive value; SES, Static Ensemble Selection; DEP, Dynamic Ensemble Pruning; Best Base, best-performing base model.

**Supplementary Table 8. Thresholds of nonlinear associations between continuous predictors and live‑birth probability derived from RCS analyses**

| Feature | Range | P_overall | P_nonlinear | Peak/Plateau Point |
| --- | --- | --- | --- | --- |
| Female Age | 24 - 45 | <0.001 | <0.001 | 29 (Peak) |
| Male Age | 25 - 51 | <0.001 | <0.001 | 36 (Peak) |
| AMH | 0.20 - 11.08 | 0.005 | 0.04 | 5.7 (Peak) |
| AFC | 1- 30 | <0.001 | 0.002 | 19 (Peak) |
| Endo Thickness | 6 - 17 | <0.001 | 0.002 | 12 (Plateau) |
| Oocytes Retrieved | 1 - 28 | <0.001 | <0.001 | 15 (Peak) |
| Cleaved Embryos | 0 - 20 | <0.001 | <0.001 | 11 (Peak) |
| High-quality Blastocysts | 0 - 8 | <0.001 | 0.012 | 4 (Peak) |
| Total Frozen | 0 - 11 | <0.001 | <0.001 | 6 (Peak) |

Peak refers to the value corresponding to maximum predicted live‑birth probability; Plateau indicates the inflection point beyond which predicted live‑birth probability remains stable.
